# Protecting the Prehospital Professional First Aid Teams from Airborne Viral Particles in the Case of Out-of-Hospital Pediatric Cardiac Arrest during the COVID-19 Pandemic

**DOI:** 10.1017/S1049023X2000062X

**Published:** 2020-05-12

**Authors:** Sabine Lemoine, Frederique Briche, Daniel Jost, Bertrand Prunet

**Affiliations:** Paris Fire Brigade, Medical Emergency Department, Paris, France

To the Editor,

The pediatric 2019 novel coronavirus disease (COVID-19) cases report mostly asymptomatic or mild infections, with a better prognosis than in adults.^1,2^ Any pediatric victim who dies from COVID-19 has a significant media impact. These pediatric COVID-19 cardiac arrests occur mostly in intra-hospital settings. To date, we have not found any publication on the prehospital management of COVID-19 pediatric out-of-hospital cardiac arrest (OHCA) by Basic Life Support (BLS) teams (personal systematic review).

In Paris, France, the Fire Brigade Prehospital Rescue System treats approximately 50 pediatric OHCA per year. From March 20, 2020 to April 7, 2020, two children benefited from prehospital cardiopulmonary resuscitation by a BLS team with an unknown etiology.

During the pandemic, in the case of pediatric OHCA whose etiology is definitely not a COVID-19 infection, children should be considered in all cases as potential carriers of COVID-19, and therefore as contaminants as a symptomatic adult, particularly from nasopharyngeal and stool excretion.^1^

We are focusing on raising awareness among prehospital teams working on pediatric OHCAs about the need to systematically protect themselves regardless of the cause of pediatric OHCA, and routinely wear personal protective equipment (gloves, filtering face piece [FFP2] respirators, goggles, and long-sleeved gown), especially since there are still uncertainties about the spread of airborne viral particles during resuscitation procedures such as chest compressions, defibrillation, insufflation maneuvers, tracheal intubation, and oral and pharyngeal aspirations.^3,4^

The International Liaison Committee will reach a consensus on the science of the balance between therapeutic benefit and risk of contamination for each of these procedures, also for pediatric cases.
